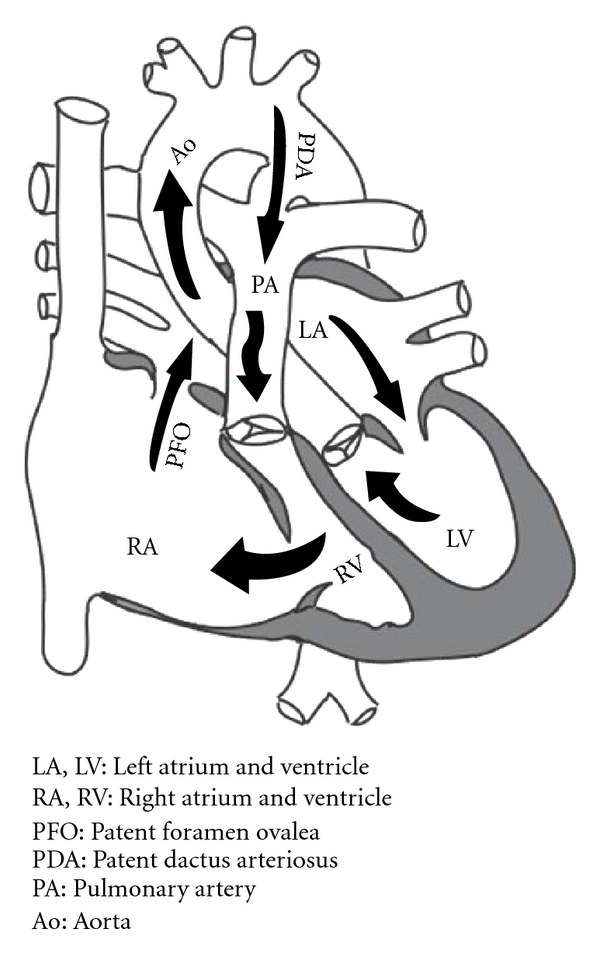# Neonatal Cyanosis: Diagnostic and Management Challenges

**DOI:** 10.5402/2011/175931

**Published:** 2010-12-29

**Authors:** A. Izraelit, V. Ten, G. Krishnamurthy, V. Ratner

**Affiliations:** Department of Pediatrics, Columbia University College of Physicians and Surgeons, New York, USA

## Abstract

Neonatal central cyanosis is always a sign of serious pathological processes and may involve diverse organs and impose a significant diagnostic and therapeutic challenge. Here, we report an unusual presentation of Ebstein's anomaly, a rare congenital heart malformation, as the cause of central cyanosis in a one-week-old full-term infant. Initiation of PEG_1_ therapy in neonates with Ebstein's anomaly always needs a very careful consideration because of a high risk for the development of a “circular shunt” leading to severe deterioration of multiple organs perfusion.

## 1. Introduction

**Figure 1:**
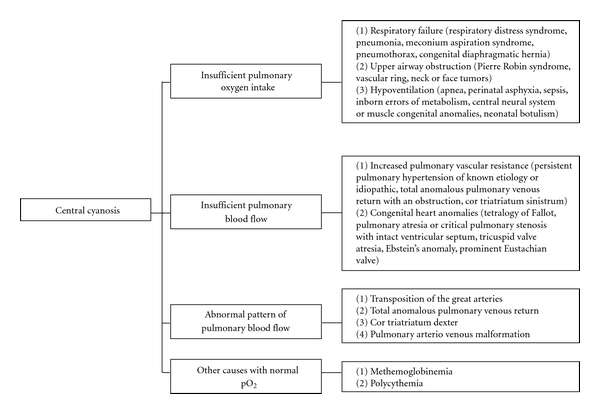


Cyanosis is a blue discoloration of the skin and mucus membranes caused by an increased concentration of reduced hemoglobin (>1.9–3.1 mmol/L) in the blood. Peripheral and central forms of cyanosis are well recognized. Peripheral or acrocyanosis in newborns is regarded as a benign transient discoloration of the hands and feet. Central cyanosis is a serious pathological sign and involves discoloration of lips and tongue. The list of the pathophysiological causes and the most common disorders summarized in Figure 1. Clearly, it can be a formidable task to reach the right diagnosis in a neonate with central cyanosis. The case presented below illustrates diagnostic and management challenges.

## 2. Case Report

A female neonate was delivered at term following unremarkable prenatal course with normal fetal ultrasound. The infant was delivered via elective cesarian section. The Apgar scores were 9 and 9 at the first and fifth minute, respectively. The birth weight was 2525 g with a normal body length and head circumference. 

**Figure 2:**
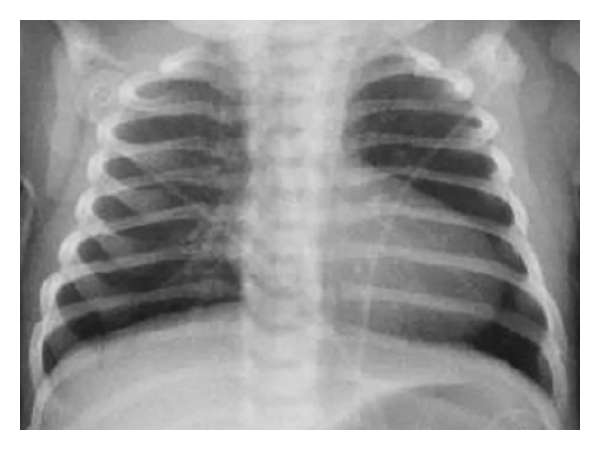


**Figure 3:**
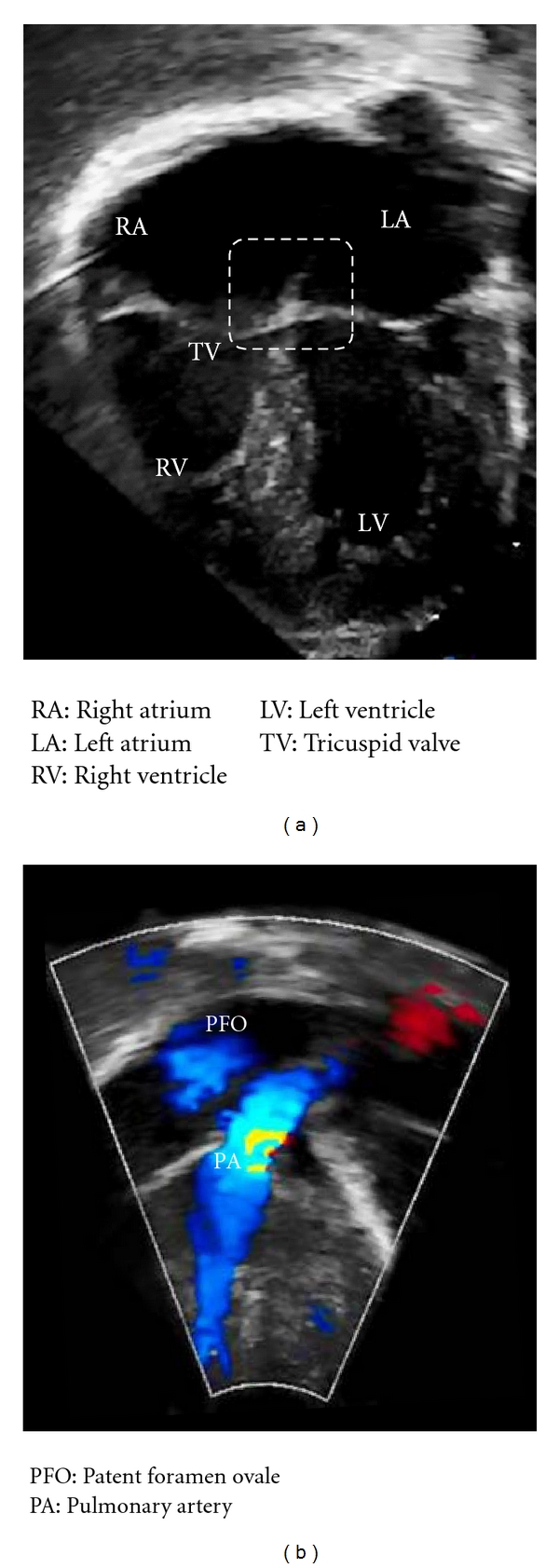


On the 8th day of life, the infant exhibited central cyanosis during crying. On physical examination, the nondysmorphic infant was found to be cyanotic, warm, and well perfused. Vital signs at that time were as follows: body temperature of 36.8°C, heart rate 193 beats/min, respiratory rate 45 breaths/min, pre and postductal oxygen saturations 77% and 81%, respectively, and blood pressure 93/45 mmHg without gradient between upper and lower extremities. Chest examination showed clear breath sounds bilaterally without retractions. Cardiovascular examination revealed quiet precordium, a regular rhythm with normal S1 and S2, no murmurs, and palpable femoral pulses. Abdomen was soft without hepatosplenomegaly or masses. The muscle tone was appropriate for age. Arterial blood gas sampled from the right wrist on room air demonstrated a pH of 7.39, pO_2_ of 42 mmHg, pCO_2_ 32 mmHg, and arterial lactate of 8.7 mmol/L. The infant failed the hyperoxia test on CPAP FiO_2_ 1.0 (pO_2_ 66 mmHg). Sepsis evaluation (complete blood count, C-reactive protein, and blood culture) was performed and antibiotic therapy was commenced. Chest X-ray illustrated a mildly enlarged boot-shaped cardiac silhouette with absent pulmonary artery shadows, oligemic lung fields, and no focal pathology (Figure 2). Electrocardiogram (EKG) showed normal sinus rhythm, QRS axis of +20°, and no right ventricular forces in V1-V2. Transthoracic ECHO revealed an unobstructed antegrade flow into the hypoplastic right ventricle and outflow without evident restriction or insufficiency (Figure 3(a)); a significantly echogenic tricuspid valve with inferior displacement of anterior and septal leaflets into the ventricle and absent tricuspid valve regurgitation (Figure 3(a)); right atrium moderately dilated with right-to-left shunting through the foramen ovale (Figure 3(b)); no flow through the ductus arteriosus. Consequently, a diagnosis of Ebstein's anomaly was made.

Prostaglandin E_1_ (PGE_1_) therapy was not initiated due to presence of forward flow through the pulmonary valve and the oxygen saturation above 80% on CPAP FiO_2_ 0.45. Arterial lactate level normalized within 24 hours from the admission, and respiratory support was gradually discontinued. The infant remained on room air with oxygen saturations in the 80 s without desaturations. Following period of stabilization, oral feeding was resumed and the infant was able to nipple *ad libitum*. The blood culture remained negative and the antibiotics were discontinued after 48 hours.

No surgical intervention was needed, and the infant was discharged home on the 4th day from the admission. Two-month followup revealed that the patient's appropriate growth and development was suitable for her age, and her oxygen saturations were in the high 80 s on room air.

## 3. Discussion

The presented case represents a common challenge in neonatology. The presence of central cyanosis in the newborn without respiratory distress, with boot-shaped cardiomegaly and failed hyperoxia test favored a diagnosis of congenital heart disease. Absence of normal neonatal right-sided forces on EKG would have likely entertained a possible diagnosis of pulmonary atresia with intact ventricular septum, tricuspid valve atresia, or Ebstein's anomaly. However, these anomalies are associated with presence of heart murmur on the physical examination. In our patient it would be impossible to correctly diagnose mild form of Ebstein's anomaly without the ECHO evaluation. The patient became cyanotic at the time of ductus arteriosus closure, which limited pulmonary blood flow [1]. Ebstein's anomaly is a rare cyanotic congenital heart disease (recently reviewed elsewhere [2–4]) with wide range of severity in time of presentation.

Management of neonates with cyanotic congenital heart disease frequently begins with PGE_1_ infusion to keep the ductus arteriosus patent. This is especially true in smaller neonatal intensive care units without in-house cardiologists. It should be noted that empirical therapy with PGE_1_ might pose a serious risk for the development of a “circular shunt” in patients with Ebstein's anomaly [5] or tetralogy of Fallot with absent pulmonary valve. Blood flow through the ductus arteriosus in these patients maintains a high pulmonary artery pressure that can exceed right ventricular pressure and cause significant pulmonary valve insufficiency (Figure 4). This increases tricuspid valve regurgitation, which further lowers right ventricular pressure and increases right atrial pressure. High right atrial pressure promotes a right-to-left shunt via the foramen ovale to the left atrium and finally to the aorta toward the open ductus arteriosus completing the hemodynamically inefficient “circular shunt.” “Circular shunts” lead to severe reduction in systemic blood flow and oxygen delivery and manifest with refractory metabolic acidosis and multiple-organ dysfunction syndrome. Therefore, prior to initiation of PGE_1_ therapy in neonates with Ebstein's anomaly, it is extremely important to verify the absence of pulmonary valve insufficiency. However, PGE_1_ should not be withheld if ECHO is unavailable. If PGE_1_ therapy is used in these patients, it is important to provide close monitoring of adequate systemic circulation (blood pressure, arterial blood gases with lactate, urine output). Prompt discontinuation of PGE_1_ infusion is necessary if the infant demonstrates signs of poor systemic oxygen delivery due to the risk of “circular shunt” [5]. If the ductus arteriosus remains patent, urgent surgical ligation of the ductus will abolish the “circular shunt”. Antegrade pulmonary blood flow may be enhanced by pulmonary vasodilators (oxygen, inhaled nitric oxide). 

In conclusion, central cyanosis in the newborn is sign of serious underlying pathological process requiring urgent detailed evaluation by neonatologist, and appropriate therapy may require a highly specialized individual approach.

## Figures and Tables

**Figure 4:**